# Relative Age Effect (RAE) According to Norm Values on Anthropometric Performance and Physical Fitness in 9–11-Year-Old Children

**DOI:** 10.3390/jfmk10010032

**Published:** 2025-01-14

**Authors:** Artan R. Kryeziu, Bujar Begu, Dana Badau, Astrit Iseni

**Affiliations:** 1 Faculty of Physical Education, University of Pristina Hasan Prishtina, 10000 Pristina, Kosovo; 2 Center of Research, Studies in Physical Education, Sport and Health-CRSPES, 10000 Pristina, Kosovo; 3 Faculty of Physical Education and Mountain Sports, Transilvania University, 500068 Brasov, Romania; 4 Faculty of Physical Education, University of Tetovo, 1300 Tetovo, North Macedonia

**Keywords:** physical fitness, birth in RAE, chronological age, percentile values

## Abstract

Objectives: The main purpose of this study is to identify the relative effect of age (RAE) according to norm values on the anthropometric performance and physical fitness of children between the ages of 9 and 11 years. The data, namely the percentiles of anthropometric parameters and physical fitness, are relevant for identifying the RAE in relation to gender and the month of birth in children. Methods: For the sample in this study, 1185 young people from Kosovo were enrolled, including 626 males and 559 females aged 9–11 years. The gathered data were assessed via the EUROFIT methodology, which takes into account comparisons based on the quartile of birth and the relative age effect (RAE). Results: The results of the data show us that there are significant differences in the RAE among children born in Q2 at the age of 9 years, especially in boys, as well as significant differences in the height variable at the <0.005 significance level. Others factors did not show significant differences, for example, variables that are indicators of physical fitness. For both boys and girls born in Q1, significant differences were mainly found in the indicators of explosive strength, flexibility, static strength, speed, and agility, with a level of significance of *p* < 0.001, while other indicators did not show significant differences. In addition, there was a significant difference the ratio between genders at the *p* < 0.001 and *p* < 0.005 levels, mainly among those born in Q1 and Q2. Similarly, at 10 years of age, children who were born earlier in the year had better scores, although the large disparities were more noticeable between quartiles than between genders, where the significant differences were mainly in the indicators of explosive strength, speed, and strength at the *p* < 0.001 level, as well as static strength, agility, and speed at the *p* < 0.005 level. Significant differences were also found for the indicators in terms of gender. At the age of 11, significant percentiles were mainly found in the quartiles at the beginning of the year for both boys and girls, and significant differences were also found at the *p* < 0.001 level for RAE between quartiles and gender. Conclusions: By using these data, it will be possible to highlight how males, who have demonstrated notable advantages in anthropometric and physical fitness measures, as well as those born in the first few months of the year, exhibit a relative age effect (RAE) in accordance with gender norm values.

## 1. Introduction

In any sport activity, contemporary trends and development are closely interconnected. The level of physical fitness significantly improves between the ages of 9 and 11 [1]. This is supported by reports indicating that there are enhancements in physical fitness among different age groups, as measured by anthropometric characteristics and overall physical fitness [1,2,3]. When presenting the results of anthropometric characteristic studies and physical fitness tests, it is crucial to refer to normative values on the basis of sex and age parameters. Research has consistently shown that males generally achieve higher strength scores than females and that strength increases significantly in both genders as they grow older [4,5,6,7]. According to longitudinal research on the development of various fitness components, children between the ages of 9, 10, and 11 have performance trajectories that are gender-specific [1]. The current study, which examined four aspects of physical fitness at four annual evaluations, was a predecessor to this cross-sectional study [1,8]. To enable a comparison with the EUROFIT data, gender- and age-specific normative values for European children and adolescents (aged 9–17 years old) were generated in this study by assessing over 2.5 million cases gathered from 98 studies in 30 European nations [9]. The following section describes the physical fitness standards for Portuguese children and adolescents on the basis of their age and gender. A previous study analyzed many physical fitness markers and reported that males consistently outperformed females in all fitness tests [2]. It has been suggested that age (specifically chronological age) might have a negative impact on an individual’s future participation in a sport, particularly if individuals are born in the early or late months of the year [10]. One explanation for the recent focus on scientific data is the relative age effect (RAE), which refers to the tendency to favor prospective players born in the early months of the year during the selection process [11]. The relative age effect (RAE) has been reported in competitive contexts when participants are stratified on the basis of chronological age [12]. One study demonstrated that the relative age effect (RAE) is commonly detected when individuals are categorized on the basis of their chronological age and anthropometric status. Additionally, a previous study revealed that experience and opportunities for practice differ considerably with age [12]. When we refer to the relative age effect (RAE), our focus is on the inclusion of anthropometric characteristics, physical fitness, and the social, behavioral, and limiting factors of the environment and the social circle of the children included in the studies [13,14,15], as well as the variations in socioeconomic status. It is important to identify how these factors contribute to the performance of children as quickly as possible [16], hence the need to include more relevant indicators. A validated study in Slovenia investigating anthropometric characteristics, physical fitness, and environmental and socioeconomic components in relation to children suggested that improvements in their socioeconomic status led to increased physical fitness in the population [17,18]. Low socioeconomic status (SES) has been associated with lower levels of fitness [19,20], fewer intentions to engage in healthy behaviors [21,22,23], and adverse health outcomes.

The EUROFIT protocol was used to conduct this investigation. The results of this study can be used to assess the disparities in chronological age on the basis of birth dates between males and females using separate standards. In our country, there is limited research on the relative age effect (RAE) and norms for individuals aged 9–11 years. However, even in other countries, few studies have investigated the influence of sex and RAE on anthropometric characteristics and physical fitness tests in 9- to 11-year-olds. Hence, the objective of this study was to examine the influence of birth month, which is based on established standards, on the anthropometric characteristics and physical fitness of children between the ages of 9 and 11. Additionally, another objective is to examine the disparities in anthropometric measurements and physical fitness between males and females on the basis of age, as well as the variations among individuals born in different months, in accordance with established standards.

We aim to investigate two hypotheses in our study. The main hypothesis is that there will be a significant increase in RAE based on norms of gender and age in 9, 10, and 11 years. To investigate this hypothesis, first, we assume that the anthropometric characteristics and physical fitness of the children in relation to gender and age can be presented as percentiles. Second, we assume that the data, presented as percentiles between age and gender, have significant differences between birth months. Our second hypothesis is that children born earlier in the year will have higher scores on physical fitness components than those born later in the same calendar year.

## 2. Materials and Methods

### 2.1. Study Design

This study outlines the EUROFIT test protocol as a study design, examining the impact of birth month on anthropometric characteristics and physical fitness in children aged 9–11 years on the basis of established norms. It also examines the disparities between sex and age regarding the standards of anthropometric markers and physical fitness. Therefore, this study focuses on elaborating on the experimental research methodology, utilizing data from children aged 9, 10, and 11 years old, interpreting the general meaning of the results, and, as a cross-sectional study, identifying the relationship between age and gender, including percentile values between quartiles and significant differences across studies. In the framework of this study, only children aged 9, 10, and 11 years old were selected based on a standard test protocol according to the normative values defined to obtain the relevant data on gender, age, and RAE.

### 2.2. Participants

This section will discuss the various phases and stages of the participants’ involvement in the testing process for physical fitness assessments. For this research case study, we selected students who did not participate in extracurricular activities. This study encompassed a total of 1185 children, predominantly males and females between the ages of 9 and 11 who attended 15 schools in Kosovo. Considering the overall sample, there were 397 individuals aged 9, consisting of 213 males and 184 females; there were 378 individuals aged 10, with 195 males and 183 females; and, finally, there were 410 individuals aged 11, comprising 218 males and 192 females. Considering the complete dataset, there were 626 male participants and 559 female participants.

In order to include the participants in this study, several stages of sample selection were carried out. Ethical approval of the research was first obtained from the local Ethics Committee for Research at the University of Business and Technology (UBT). This approval was granted in accordance with the Helsinki Declaration on Biomedical Research with Humans (WHADH, 2000) (Approval 12897/45). Approval was also received from the Ministry of Education, Science and Technology (MASHT) (Approval 3/158-2), the Municipal Director of Education (DKA), and the schools from which the children were selected. The study took place in Kosovo, which has a total population of >1,585,566, comprising 795,552 males and 790,014 females across eight cities/municipalities: Pristina (population of 227,466), Prizren (population of 147,246), Ferizaji (population of 109,255), Gjilani (population of 82,980), Mitrovica (population of 64,742), Podujeva (population of 70,975), Fushë Kosova (population of 63,949), Suhareka (population of 45,749), and Drenas (population of 48,079). In our final report submitted to the Ministry of Education, Science and Technology (MASHT), we discussed the challenges of representing this whole population and the lack of access that the research team of the Center of Research, Studies in Physical Education, Sport and Health (CRSPES) had to schools in all municipalities. These parts of the population represent a large population in terms of settlements within these cities, so the density of the population in these settlements and the gender ratio in terms of chronological age, as well as the number of autochthonous/native participants, may not be representative of the total population of children from the city and the regions they come from [24,25,26,27]. Before starting the testing, the participants were informed about the study methodology, the purpose, and the criteria, as well as the methods used to conduct the tests, by their guardians, and parental consent for their testing was obtained via a written confirmation form. The children’s consent was also obtained via a written consent form, and before the test, they were asked verbally to confirm their participation in the research. Before starting the test, participants were encouraged (1) to familiarize themselves with the process of the study and the possible risks that could arise during the implementation of the test. (2) Participants also had to confirm that they had not experienced any injury or damage before the start of the study and (3) complete a form for reference during the study before completing the test. (4) Participants were also told to wear adequate sports clothing throughout the experiment and (5) were assured that the confidentiality of their data and the results would be preserved during and after the end of the study, with the data only being published for the purpose of the study [18]. Since its establishment over 10 years ago, the research team of the Center of Research, Studies in Physical Education, Sport and Health (CRSPES) has continuously managed to create useful methods of testing and follow-up monitoring of various parameters and programs. It should be noted that before the test, a workshop was held with the entire research team to discuss methods for testing the relevant parameters presented in this study and the progress in testing with technological equipment, as is the case for speed evaluation using the WITTY photocell system from Microgate, ITA [28], as well as Microgate’s OptoJump system [29]. Also, during the execution of the work, the passes (which happened the day before going to the field), testing, and valorization with subsequent tests were carried out with the aim of having as few errors as possible or trying to avoid any complete or occasional omissions in the data. For example, when taking measurements, the data were recorded on log sheets that were then placed in a special bag to protect them from possible damage such as moisture or air compression. After each day in the field, detailed information about the work, its progress, and any possible releases was recorded, aiming to avoid any release on other days of the test, and the data were finally entered into the database. The research team also checked and filtered the database before the testing phase with relevant statistical methods [24,25,26].

### 2.3. Test Protocol

#### 2.3.1. Anthropometric Measurements

Body mass and height were assessed via established protocols [30]. The height of the participants was measured via a stadiometer (Model 220 SECA, Hamburg, Germany) with an accuracy of 0.1 cm. Their mass was measured via an electronic scale (TANITA model SC-240MA) with an accuracy of 0.01 kg. The measurements were taken when the participants were wearing minimal clothing and no shoes. Body mass index (BMI) was computed by dividing body mass by the square of height (kg/m^2^). The measurements were conducted under identical test conditions and durations. The methodology and interpretation of the anthropometric measurements have been previously detailed in other publications [31].

#### 2.3.2. Measurement of Physical Fitness

The physical fitness tests were chosen from the EUROFIT test protocol on the basis of prior research. These tests are utilized to assess various aspects of children’s physical fitness, including balance, flexibility, explosive strength in the lower limbs, speed and running ability, endurance of the arm and shoulder muscles, repetitive strength of the abdominal muscles, isometric strength of the hand muscles, and speed with agility [30]. The complete EUROFIT test regimen consists of the following:

1. Flamingo balance test. This assessment evaluates an individual’s balance. The participant is instructed to maintains equilibrium on a beam with dimensions of 50 cm in length, 4 cm in height, and 3 cm in width. Stability is ensured by two supports that are 15 cm in length and 2 cm in width. The beam is positioned in front of the young person. They are permitted to have a practice run to become acquainted with the test, which includes receiving pertinent instructions. Following this practice, the testing begins. At the instant when the participant removes their arm from the support, the stopwatch starts to measure time. The chronometer is halted when the person loses balance, releases the leg, or makes contact with the ground using any part of their body [30,32,33]. The flamingo balance test showed high values for validity and reliability between 0.84 and 0.98 [34]. After completing the test, the individual is evaluated by determining the number of attempts required (excluding any instances of falling) to achieve a state of balance.

2. Sit and reach (cm). Firstly, the chest of the participant is measured with a centimeter tape. This test requires the participant to take a seated position on the floor with their legs extended straight in front of them. It is necessary to take off sports shoes (sneakers). The plantar surfaces of the feet are situated in proximity to the enclosure. Both knees should be flexed and managed. The participant extends their arms forward along the measuring line, with their palms facing downward and hands either stacked on top of one another or placed side by side. It must be ensured that both hands are maintained at a consistent level and one does not extend further than the other. Once the participant has become acquainted with the task, they move into the desired posture and maintain it for a brief period of one to two seconds, during which the distance is measured and documented [30]. The sit-and-reach test proved reliable with a test–retest reliability coefficient between 0.73 and 0.96 among the children [35]. The outcome is measured to the closest centimeter and documented in the log book.

3. Standing long jump (cm). The objective of the participant is to use the maximum force of their legs to propel themselves as far as possible while maintaining a straight body position and landing with their legs close together. The evaluation is conducted by quantifying the leap’s length, namely, the distance between the starting line and the final mark following the jump [30]. The SLJ hank showed adequate values for validity and reliability ranging from 0.52 to 0.78 and 0.66 to 0.97 [36,37]. Three jumps are performed, and the best outcome is documented with a precision of 0.1 cm and recorded on the record sheet.

4. Countermovement jump (CMJ). The height of the jump is calculated via a time table. In this case, we apply OptoJump, which measures the time when the legs are out of bounds. The child stands up straight in light sneakers as quietly as possible on a mat, with their weight evenly distributed between both feet. Their hands must be placed on the hips and remain there during the entire test. When everything is ready, the child lowers themself down until their knees are bent at a 90° angle and then immediately jumps vertically as high as possible, landing again on the floor on both feet at the same time. A suitable period of rest should be taken between attempts. The lift should be from both legs, without initial steps or movements. They also should not stop at the base of collection [38,39]. The CMJ showed high values for validity and reliability with 0.87 for internal consistency and a Cronbach’s α of 0.98 for reliability [40]. The best result of at least three attempts is recorded on the score sheet.

5. Handgrip strength. This test is an assessment of isometric strength. The subject has their legs slightly extended, and a dynamometer is placed close to the toes. Their arm is placed down along the body but without touching it. They then quickly grip the dynamometer using maximum force while the other arm is loose along the body. Handgrip strength showed adequate values for validity and reliability ranging from 0.78 to 0.85 [41]. The best results of the three tests are recorded; the result is rounded to the nearest 1 kg [30].

6. Sit ups. This test is used to evaluate the strength and endurance of the abdominal muscles. The person assumes a seated position with their hands placed behind the neck and crossed. The legs should be partially folded, and the body should be fully extended while the subject maintains a fixed position with their legs. The individual must swiftly elevate the barbell from the ground to a vertical position repeatedly within a time frame of 20 s. The quantity of vertical lifts is counted, and the meter regulates the magnitude of motion [30,42,43]. The sit up test showed adequate values for validity and reliability ranging from 0.78 to 0.97 [44]. After three attempts, the highest attained result with an accuracy of 0.1 is recorded.

7. The 20 m sprint. This test is carried out to evaluate speed. A distance of 20 m is measured from the starting line and set as the target line. The participant runs at their greatest velocity toward the finish line, and the run is timed via a Microgate timer. The Microgate instruments of the Witty SEM system [30,37] are used for electronic time measurements. The 20 m sprint has an adequate value for validity and reliability of 0.93 [45]. The best outcome was documented after two trials, with an accuracy of 0.1 s, and was entered into the sheet registry.

8. Bent arm hang. This test is an assessment of static strength, specifically the endurance of the arm and shoulder muscles. The participant keeps their arms crossed while hanging clothes on an ironing board. A 2.5 cm diameter iron rod is positioned horizontally on the ground, allowing the subject to easily grip it without needing to jump. Initially, the researcher positions the subject beneath the iron, ensuring that their hands pass over it. The thumb is placed below and in front of the iron at a distance equivalent to the width of the subject’s shoulders. A stopwatch is grasped by one hand, while the other hand supports the subject’s thighs, elevating them to the desired position. Currently, the chronometer is activated, and the subject is not allowed to leave until their chin rests on the iron. If a swinging motion occurs, the patient is prevented from continuing. The stopwatch is halted when the subject is unable to sustain the position or when their eyes descend below the level of the iron [30]. The bent arm hang has an adequate value for validity and reliability of 0.99 [46,47]. After two trials, the highest achievable result with accuracy of 0.1 is documented and registered in the sheet registry.

9. The 10 × 5 m shuttle run. Initially, two parallel lines are marked on the ground (with chalk or strips) 5 m apart. These lines must be 1.20 m long and be connected at their exteriors to boxes, cubes, corners, bottles, etc. During the test, care is taken to ensure that the subject crosses the remaining line on the road track with both feet after performing the half-turn, even at high speeds. After each cycle, the number of completed cycles is announced aloud. The test is completed when the subject crosses the finish line with only one foot. The child should not slip while running [30]. The 10 × 5 m SR test has adequate values for validity, ranging from 0.62 to 0.85, and reliability, ranging from 0.62 to 0.96, in children [48]. The recording time for all 5 cycles is recorded with an accuracy of 0.1 and recorded on the log sheet.

### 2.4. Statistical Data Processing

This research study utilized field-collected data as the foundation, and appropriate procedures for data processing were chosen. On the basis of anthropometric measurements and physical fitness tests conducted on males and females aged 9–11 years, we calculated the following statistical measures: arithmetic mean (Mean) and standard deviation (Std.Dev). We also identified significant differences between sex and age via univariate analysis of variance (ANOVA) with Scheffe’s model at the probability levels of 0.001 (**) and 0.005 (*). Percentages ranging between very poor (5%, 10%), poor (25%), average (50%), good (75%), very good (90%), and excellent (95%) were determined on the basis of sex and age groups of 9, 10, and 11 years. Additionally, graphics illustrating anthropometric factors and physical fitness tests have been included and processed with the JAMVOI program (version 4 MacOS) using the universal analysis of covariance (ANCOVA) with Scheffe’s model at the probability level of 0.005 (*). The study also utilized the relative age effect (RAE) model, where the months of birth of the children were divided into quartiles (Qs). The annual calendar is considered to cover the period from January 1 to December 31. The first quartile (Q1) includes the months of January, February, and March. The second quartile (Q2) includes the months of April, May, and June. The third quartile (Q3) includes the months of July, August, and September. Finally, the fourth quartile (Q4) includes the months of October, November, and December. After the event, the collected statistical data were evaluated and processed via the statistical program SPSS version 26, which yielded the obtained results.

## 3. Results

Table 1 contains the anthropometric performance data and physical fitness test results categorized by sex and age. Anthropometric characteristics include measurements of height, body mass, and body mass index. At the age of 10, both sexes have the same height values. However, at the ages of 9 and 11, there are more noticeable differences in height between the sexes, which are considered normal in these circumstances. Body mass demonstrated significant sexual dimorphism, with higher values observed. However, at the age of 9, the body mass index indicates that most children are underweight, whereas 10- and 11-year-olds exhibit weights within the normal range. The physical fitness indices notably increased with increasing age in both male and female individuals. Specifically, the findings of the sit-and-reach and flamingo balance tests revealed a decline in performance as individuals aged, regardless of sex. The standing long jump and countermovement jump (CMJ) tests exhibited a gradual improvement in results with the age of the children. The 20 m sprint test demonstrated an important correlation between age and performance, as did the 10 × 5 m shuttle run, where better results were observed with increasing age. The bent arm hang test results demonstrated the advantages experienced by males at the age of 9.

In Table 2, percentages of significant differences between children aged 9 born in Q1, Q2, Q3, and Q4 were determined by analyzing the relative effect of age (RAE) and gender differences in each quartile individually. The females had higher values for the height variable, specifically in Q1. On the other hand, males had higher values in the other birth quartiles. However, when comparing the birth quartile system as a whole (Q1, Q2, Q3, and Q4), there are significant differences in percentages (*p* < 0.005 (*)). When looking at each quartile separately (Q1, Q2, Q3, and Q4) by gender, there are no significant differences. When considering body mass variables based on percentages, females presented higher values than males did. Specifically, when examining the changes in each quartile separately, we observe that in Q3, there are higher values for both sexes. However, there were no significant differences between the two sexes. The body mass index variable, as per the established standards, yields a combination of results. However, when examining the quartiles individually on the basis of %, we observe that in the second quartile (Q2) at birth, both sexes present greater values. Nevertheless, there are no significant differences between the sexes in this regard. The forward trunk fold test, when analyzed by quartile, revealed that individuals in Q3 at birth presented higher values in both males and females. However, the differences are more pronounced in males. When the overall quartile system is considered, significant differences are observed (*p* < 0.015 (*)), but no significant differences are found between the two sexes. Males demonstrated superior performance in the standing long jump test when measured as percentages. Females showed exceptional results in the fourth quartile of birth dates, whereas males excelled in the third quartile. In the overall quartile system, we observed significant differences in values (*p* < 0.001 (**)), as well as significant differences between males and females (*p* < 0.001 (**)). Countermovement jump (CMJ) favored a greater proportion of males than females. In the overall quartile system, we observe significant disparities (*p* < 0.000 (**)), and there are also notable distinctions between males and females (*p* < 0.000 (**)). Regarding the flamingo balance test, we observe a percentage distribution between males and females. However, it is worth noting that males achieved superior results compared with females on the basis of percentage. In the general quartile system, we did not observe any significant differences. In the 20 m sprint test, males presented higher values than females when percentages were considered. Females born in Q3 performed the best, whereas males born in Q1 particularly excelled. Overall, there were significant differences (*p* < 0.000 (**)) among quartiles based on birth month, but no significant differences were detected between females and males. The abdominal muscle test revealed that a greater percentage of males, particularly those born in Q3, excelled in the task than females. On the other hand, the highest-performing females were those born in Q3 and Q4. When the overall system was considered, there were significant differences in the general values (*p* < 0.000 (**)) and significant differences between males and females (*p* < 0.000 (**)). After conducting a 10 × 5 m shuttle run test, we analyzed the results on the basis of quartiles. It was observed that males born in Q3, Q1, and Q2 achieved better results than females. Specifically, males born in Q3 performed the best, followed by males born in Q1 and Q2. On the other hand, females born in Q3 performed better than females born in Q1, and females born in Q1 performed better than females born in Q2. When the overall system was considered, significant differences in value were observed (*p* < 0.000 (**)). Additionally, when the system was analyzed separately, significant differences in value were found between females and males (*p* < 0.000 (**)). The bent arm hang test results indicated that males born in Q3 and Q1 had higher percentages, whereas females born in Q4 had higher values. In the overall system, there were significant differences in values (*p* < 0.000 (**)). When the system was considered separately, there were significant differences between females and males (*p* < 0.004 (**)). Males born in Q1 presented higher values than females in the handgrip strength test, according to the percentages. However, males born in Q2 had better values. Importantly, there are significant differences in the values between those born in Q1, Q2, Q3, and Q4 for both sexes (*p* < 0.000 (**)).

As shown in Table 3, at the age of 10, significant disparities in percentages were observed among participants born in Q1, Q2, Q3, and Q4, as well as between genders within each quartile. The highest values for the variables of height, body mass, and body mass index were primarily associated with Q1, although no significant differences were detected among them. When comparing standing long jump performance between males and females, we found that males generally had better scores. Additionally, there were significant differences in performance between different quartiles, with a *p* value of less than 0.000. Furthermore, there were significant differences in performance between genders, with a *p* value of less than 0.000. The countermovement jump (CMJ) test shows higher values in males born in Q4 and females born in Q4. Males born in Q2 and females born in Q3 also had relatively high values. However, there were no significant differences between the sexes. The 20 m sprint test demonstrated higher percentage values in Q1, Q2, and Q4 for males and in Q3 for females. However, when the total system was considered, there were significant variations in values (*p* < 0.000 (**)) and substantial disparities between genders (*p* < 0.000 (**)). The abdominal muscle test revealed a greater percentage of data suggesting excellent performance in males, particularly among those born in Q2, Q3, and Q4. In the general system, these quartiles presented significant differences in value (*p* < 0.000 (**)). Furthermore, when males and females were compared, there were also significant value differences (*p* > 0.000 (**)). For the test involving a 10 × 5 m agility shuttle run, the results revealed a greater success rate in females born in Q1 than in those born in Q2. Additionally, males born in Q1 were found to dominate, with other quartiles following suit. When the overall system was considered, significant differences were observed, with a value of *p* < 0.003 (*). Furthermore, when the system was examined separately, there were no significant differences between females and males born in Q4. The bar graph shows that males born in Q2, Q3, and Q4 have higher dominance values than females. In the overall system, there are significant differences in values (*p* < 0.003 (**)), indicating a notable distinction. Similarly, in the separate system, there are substantial differences in values (*p* > 0.012 (*)). When the handgrip strength results are compared, males born in the latest quartile (Q4) have a better value, whereas for females, those born in the earliest quartile (Q1) have a better value.

Percentages and notable disparities among 11 year olds born in Q1, Q2, Q3, and Q4, as well as between genders within each quartile, are shown in Table 4. When the height variable was analyzed, it was observed that females tended to have higher values than males, especially those born in Q2. On the other hand, males born in Q3 had higher values. However, when considering the overall system of quartiles (Q1, Q2, Q3, and Q4), there were no significant differences between them. Additionally, when each quartile was examined separately (Q1, Q2, Q3, and Q4) by sex, there were significant differences (*p* < 0.009 (*)). Being born in Q3 results in greater values for anthropometric characteristics such as body mass and body mass index (BMI). In the remaining quartiles, we observe a mixture of values, with no significant difference between quartiles or between genders. During the sit-and-reach test, we observed a noteworthy proportion of females born in Q4 excelling in the test, but the values for males were comparatively lower. Additionally, we found significant disparities both across the quartiles and between the sexes, with a *p* value of less than 0.008 (*). The standing long jump test shows a higher value in males, particularly those born in Q4. There are also lower values in other quartiles for both males and females. Significant differences are observed in these values, with a *p* value of less than 0.000 (**) for both genders. In the CMJ test, males born in Q2 presented the highest percentage values, while the other quartiles also included females. However, there were significant variations in value (*p* < 0.000 (**)) both across genders and within each quartile. The results of the flamingo balancing test in Q2 demonstrated substantial values. However, when considering the other quartiles, it becomes apparent that there are no significant differences observed in this test. Compared with the other quartiles, males born in Q1 and Q2 achieved higher percentages in the 20 m sprint test. However, upon closer examination, no significant differences were observed in this test. An analysis of the results of the sit up test revealed that males born in Q4 and in subsequent quartiles achieved higher scores than females. Furthermore, there are significant differences in values between the quartiles (*p* < 0.000 (**)) and between genders (*p* < 0.000 (**)). The proportion of males born in Q1 who excelled in the 10 × 5 m shuttle run test was greater than those born in the other quartiles. When we analyzed the differences between quartiles, we observed significant variations in value (*p* < 0.000 (**)). Additionally, there were significant differences between sexes (*p* < 0.000 (**)). The results of the bent arm hang test for males were high among those born in Q1, whereas lower values were observed among those born in the other quartiles. However, no significant differences were found in this test. The best results for the handgrip strength test results among females fell within Q4, whereas the males’ results were lower in each quartile. However, it is important to note that there were no significant differences observed between the two groups in this test.

**Figure 1:**
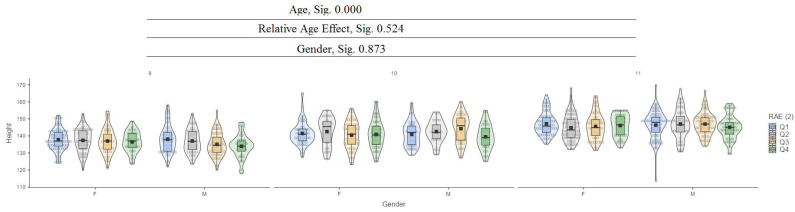
Height.

Figure 1 presents the relative age effect (RAE) between the ages of 9 and 11 years and gender.

For the height variable, it can be seen that between ages (9, 10, and 11), there are significant differences in values at the 0.000 level, while for the relative age effect (RAE) and gender, there are no significant differences (Figure 1).

**Figure 2:**
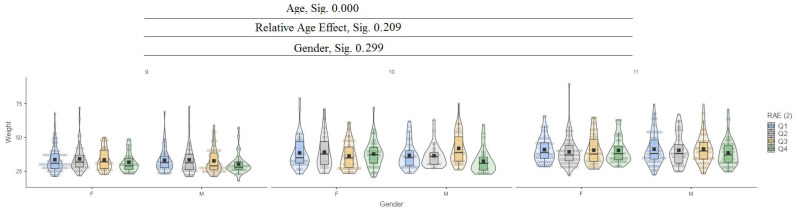
Weight.

For the body mass variable, it can be seen that between ages (9, 10, 11), there are significant differences in values at the 0.000 level, while for the relative age effect (RAE) and gender, there are no significant differences (Figure 2).

**Figure 3:**
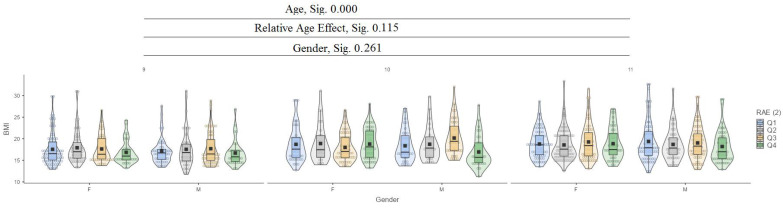
Body mass index (BMI).

In the body mass index (BMI) variable, it can be seen that between ages (9, 10, and 11), there are significant differences in values at the 0.000 level, while for the relative age effect (RAE) and gender, there are no significant differences (Figure 3).

**Figure 4:**
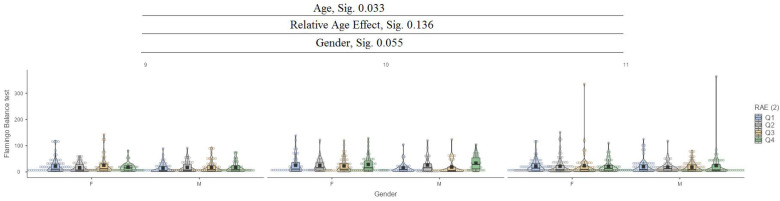
Flamingo balance test.

In the flamingo balance test, it can be seen that between ages (9, 10, and 11), there are significant differences with a low value of 0.033, while for the relative age effect (RAE) and gender, there are no significant differences (Figure 4).

**Figure 5:**
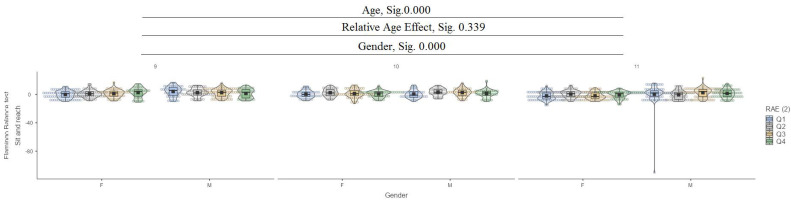
Sit and reach.

In the sit-and-reach test, it can be seen that between ages (9, 10, and 11) and between genders, there are significant differences at the 0.000 level, while for the relative age effect (RAE), there are no significant differences (Figure 5).

**Figure 6:**
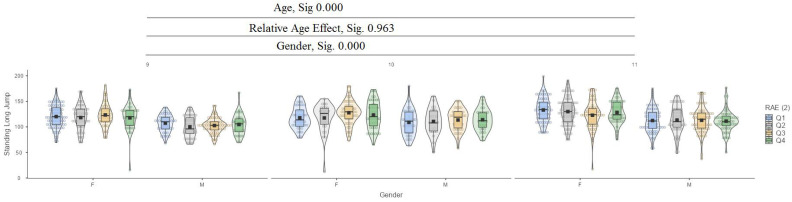
Standing long jump.

In the standing long jump test, it can be seen that between ages (9, 10, 11) and between genders, there are significant differences in values at the 0.000 level, while for the relative age effect (RAE), there are no significant differences (Figure 6).

**Figure 7:**
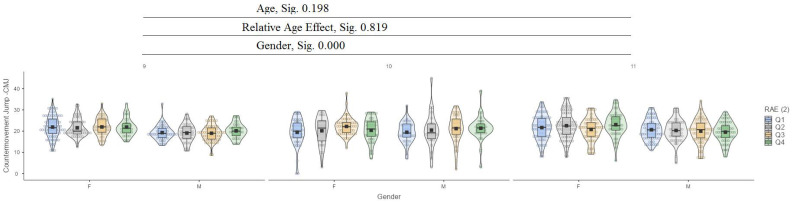
Countermovement jump (CMJ).

In the countermovement jump (CMJ) test, it can be seen that there are no significant differences between age (9, 10, 11) and relative age effect (RAE), while there are significant differences in terms of gender at the 0.000 level (Figure 7).

**Figure 8:**
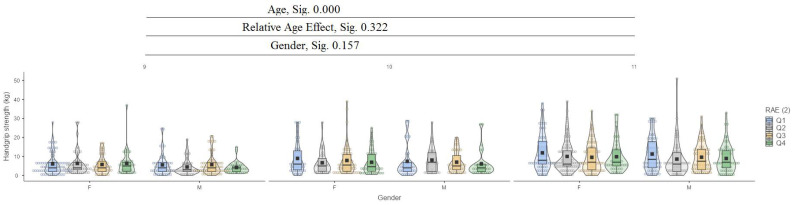
Handgrip strength.

In the handgrip strength test, it can be seen that between ages (9, 10, 11), there are significant differences at the low significance value of 0.000, while for relative age effect (RAE) and gender, there are no significant differences (Figure 8).

**Figure 9:**
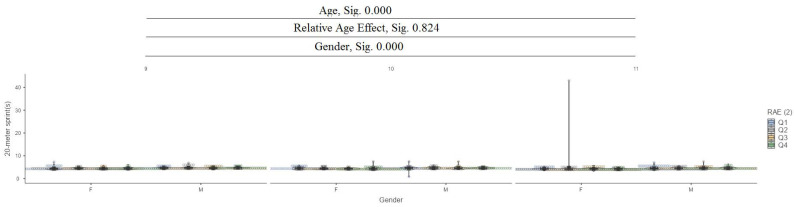
The 20 m sprint (s).

In the 20 m sprint test (s), it can be seen that between ages (9, 10, and 11) and gender, there are significant differences at the 0.000 level, while for the relative age effect (RAE), there are no significant differences (Figure 9).

**Figure 10:**
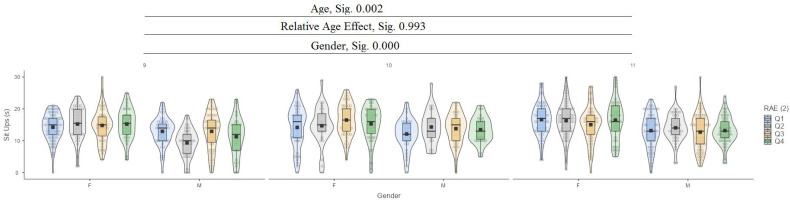
Sit ups.

In the sit up test, it can be seen that there are significant differences between ages (9, 10, and 11) at the 0.002 level and between genders at the 0.000 level, while there are no significant differences in the relative age effect (RAE) (Figure 10).

**Figure 11:**
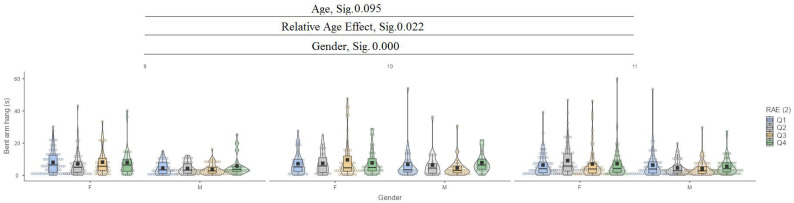
Bent arm hang.

In the bent arm hang test, it can be seen that there are no significant differences between the ages (9, 10, and 11), while for the relative age effect (RAE), there are significant differences at the 0.022 level and between gender at the 0.000 level (Figure 11).

**Figure 12:**
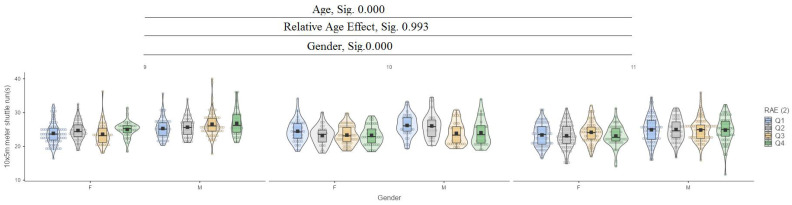
The 10 × 5 m shuttle run (s).

In the 10 × 5 m shuttle run (s) test, it can be seen that between ages (9, 10, and 11) and genders, there are significant differences at the 0.000 level, while for the relative age effect (RAE), there are no significant differences (Figure 12).

## 4. Discussion

The primary objective of this study was to examine the impact of birth month on anthropometric characteristics and physical fitness in children aged 9–11 years while also exploring sex and age differences. The discussion section covers several aspects. First, the relative age effect (RAE) between the ages of 9 and 11 years was examined. Second, it focuses on norm values on the basis of age and gender. Finally, relevant publications related to our study are presented.

### 4.1. Analysis of Body Measurements and Physical Fitness Categorized by Sex and Age

Our study revealed strong correlations between sex, age, and anthropometric and physical fitness characteristics. The parameters of height, body mass, and body mass index (BMI) exhibit optimal values at the age of 10; however, at other ages, we observe varying data patterns. The statistics from our study indicate that the age and sex groups have the lowest values in terms of height and body mass [49]. Furthermore, when we compared our findings with those of another study, we observed similar results [50]. In younger individuals, there is a gradual increase in height and body mass. However, as individuals age, there is a continuous and steady increase in both measurements. This pattern is consistent with findings from prior studies [51]. Furthermore, German writers [52] also demonstrated steady growth in terms of both age and gender. In our analysis of physical fitness indicators, we observed a notable correlation between age and sex. Specifically, we found that as individuals aged, there was a significant increase in physical fitness. However, it is important to note that the results of sit-and-reach (cm) and flamingo balance tests decrease with age and vary with sex. On the other hand, the indicator for standing long jump and countermovement jump (CMJ) shows a progressive increase in relation to both age and gender among children. In terms of chronological age, males have demonstrated more progress than females. We have corroborated data from other studies, such as the results of the flamingo balancing test carried out in [53], which demonstrate strong performance in relation to our data. Conversely, the standing long jump results from [54] demonstrate a poorer performance compared with our data. Additionally, the countermovement jump (CMJ) [55] displays progressive results in our study. The handgrip strength indicators have shown that as age increases in both genders, the data likewise increase to greater values. One study [56] supported the ratio between genders, although the results fluctuated in response to age [57]. The data analysis revealed that the abdominal muscle test, the 20 m sprint, the bent arm hang, and the 10 × 5 m meter shuttle run showed the most significant differences in performance on the basis of age and sex.

### 4.2. The Impact of Age on Anthropometric Performance and Physical Fitness, as Measured by the Norm Values for Children Aged 9, 10, and 11 Years

Our study examined the occurrence of the relative age effect (RAE) in gender-specific norms for individuals aged 9, 10, and 11 years. The reports provide normative data categorized by age and sex, specifically for very poor (5%, 10%), poor (25%), average (50%), good (75%), very good (90%), and excellent (95%) percentiles of pertinent data. The primary result of this study is the confirmation of the normative relative age effect (RAE) in both females and males born in Q1 and Q2. The study revealed that these quartiles presented more pronounced values in relation to their age. This finding is consistent with a previous study [58] that also reported the relative normative values of children in physical fitness in the same quartiles. Importantly, adjusting the data to account for sex and age has a direct effect on the relative effect of age (RAE) [59,60,61,62,63]. Our results indicate and validate our findings that at the age of 9, those born in Q1 show the highest norm values in the parameters of height, standing long jump, countermovement jump (CMJ), 20 m sprint, and bent arm hang. However, in a study conducted by Roberts S. et al. [55], it was found that other quartiles in these tests had higher normative values. Additionally, not all tests showed significant advantages in the same quartiles, taking into account age and sex. Compared with females, males presented greater values in this scenario. This is also reflected in [64], where normative data were also observed. In those born in Q3, the highest data values were observed for the standing long jump test, flamingo balance test, sit up test, 10 × 5 m shuttle run test, and bent arm hang test. On the other hand, the body mass parameters and body mass index (BMI) were greater in those born in Q2. These differences are more pronounced for males than for females. According to one study, those born in Q1 presented greater values than those born in the other quartiles [65]. Participants born in Q1, Q2, Q3, and Q4 showed significant differences, primarily in physical growth, explosive strength, repetitive strength, static strength, and fast agility. However, there were no significant differences in physical development, body mass index (BMI), balance, or isometric strength of the hand muscles. When gender differences were considered, there were significant differences in the explosive strength, balance, repetitive strength, agility, static strength, and isometric strength of the hand muscles. On the other hand, there were no significant differences in growth, physical development for BMI, or speed. Moreover, studies examining the relative age effect (RAE) on different sexes revealed notable disparities in anthropometric markers and physical fitness [65,66]. Males presented values superior to those of females in this scenario. Among those born in Q3, the highest scores were observed in the standing long jump, flamingo balance, sit up, 10 × 5 m shuttle run, and bent arm hang tests. On the other hand, the body mass parameters and body mass index (BMI) were greater in those born in Q2. These differences are more pronounced for males than for females. Only the males’ 20 m dash was improved in those born Q1. Compared with females, males exhibited superior performance in markers such as muscular strength, muscular power, muscular endurance, speed, and agility but performed worse in the flexibility test [9]. One study revealed that males outperform females in terms of academic performance. The study also revealed that age has an impact on performance, particularly in relation to the growth of upper limbs, which shows a considerable increase at the age of 10 [54]. As we age, there is a tendency to focus on data that support the physical fitness of 10-year-old males more than females. This is particularly evident in tests measuring handgrip strength and standing long jump. However, a study conducted by authors from Lisbon [67] reported a similar gender ratio. The results we examined indicate that handgrip strength values are lower than those reported in previous studies [68]; however, the sit-and-reach data demonstrated the most significant normative values in our study. In addition, our study’s data indicate lower values for both males and females in terms of handgrip strength and standing long jump indications [69]. When examining the relative age effect (RAE), we observed that among those born in Q1, there was a significant difference in outcome in the sit-and-reach (cm) test among participants of the same age. In the countermovement jump (CMJ), we find significant values for those born in Q4. Additionally, with respect to handgrip strength, we observe more significant values among those born in Q1 for both males and females [63,70,71]. Upon closer examination of participants born in Q4, it is evident that they exhibit more prominent results in handgrip strength indicators and other physical fitness tests, regardless of age and sex [72]. However, there were no significant differences in the indicators of growth and development, body mass index (BMI), flexibility, or static balance. The authors’ study revealed no significant differences in growth or physical development indicators compared with chronological age. However, children born in Q2 presented higher values for physical fitness indicators, and the only indicator that showed significant differences between the quartiles was flexibility. A study was conducted to assess physical growth, development, and fitness. The results indicated that there were no differences among the quartiles in terms of physical growth and development. Additionally, no significant differences were found in the results of the study for the other age groups. Nevertheless, there were noticeable variations in the results based on sex and age. However, no substantial disparities were observed between sex and age within the quartiles [73,74,75]. The highest percentage of rates for males in Q1 for the 20 m sprint, 10 × 5 m shuttle run, and bent arm hang tests were found at the age of 11 years. On the other hand, for females born in Q3, higher normative data values were observed for body mass parameters and body mass index (BMI) at this age. Upon closer examination of the data for those in Q4, it is evident that they present more prominent values for handgrip strength indicators and other physical fitness tests, both in terms of age and sex [72]. Furthermore, when the height of females born in the second quartile was examined, more prominent outcomes were observed than for other data. Additionally, if we consider the second quartile, we observe results in terms of body mass indicators and body mass index (BMI). In the third quartile, the sit-and-reach indicators are evident, whereas in the fourth quartile, only the handgrip strength test results are greater in females. Moreover, when the relative age effect (RAE) is analyzed, significant values are observed, indicating that these quartiles display noteworthy results in relation to sex. However, in the case of males, there is a significant difference, and the values favor males at this age. This distinction begins in the first quartile with the running test, followed by the 10 × 5 m shuttle run and bent arm hang. Furthermore, in the second quartile, the focus shifts to the counter movement jump (CMJ) and 20 m sprint. The third quartile also includes the 20 m sprint, whereas the fourth quartile involves only sit ups. The relative age effect (RAE) is observed in Q1, Q2, and Q3 according to sex at this age, as confirmed by previous studies [76]. It is also important to consider children’s area of residence and their level of physical fitness in relation to their age, which was the focus of the study [77]. Moreover, previous research has shown that the relative age effect (RAE) tends to diminish as individuals grow older and varies by gender [78]. Another study revealed that the relative age effect (RAE) was more pronounced at the age of 11 than at other ages, which coincided with an increase in the level of rivalry among different age groups. However, it is important to note that this particular study focused exclusively on females [79]. Those born in Q1, Q2, Q3, and Q4 showed significant variations in physical growth, flexibility, explosive strength, repetitive strength, and speed agility, highlighted at the <0.001 (**) significance level. However, no significant differences were observed in physical development, body mass index (BMI), balance, speed, static strength, or handgrip strength. On the basis of prior research, the majority of athletes were born in the first and second quartiles. Furthermore, there were no notable differences in the impact of age on performance between genders [80]. Notably, a study on children’s physical fitness indicators revealed variations in quartiles starting at the age of 11 years and continuing at other significant ages throughout the study [58]. Significant disparities between genders are observed in terms of physical growth, flexibility, explosive strength, repetitive strength, and speed agility. However, other indicators, such as physical development, body mass index (BMI), balance, speed, static strength, and handgrip strength, do not exhibit notable differences. Furthermore, an additional study not included here has shown that the relative age effect (RAE) during childhood not only leads to a limited selection of elite athletes but also has a detrimental influence on public health [81]. In addition, while examining other research, it has been observed that children exhibit notable variations in physical fitness measures on the basis of their age and sex [82,83,84]. When examining other research, we observe notable variations in age among quartiles and genders, which are the main focus of our work [83,85,86]. The data presented graphically in the JAMVOI program show that there are significant differences among anthropometric parameters between ages (9, 10, and 11) and genders at the 0.005 significance level, while there are no significant differences between relative age effect (RAE) and gender, as well as physical fitness, such as static leg strength, flexibility, explosive strength, handgrip strength, repetitive strength, and aerobic endurance. However, we found significant differences at the 0.005 level of significance between age (9, 10, and 11) and gender, while for the relative age effect (RAE), there was a significant difference with the static strength indicator. The study does have some limitations and strengths. The primary focus of this study is to examine the significant relationship between the relative age effect (RAE) as determined by age and gender norm values. However, this study provides a comprehensive analysis of the anthropometric parameters and physical fitness levels of children from Kosovo, taking into account their age and sex. In this study, the norms for the ages of 9, 10, and 11 years were derived on the basis of the two genders in the EUROFIT tests, taking into account the relative age effect (RAE). One notable advantage of this study is its comprehensive and extensive sample, which includes individuals of all ages and genders. The study also employs a specific testing technique to examine the impact of age (RAE) throughout the entire country. Furthermore, this study is unique in that it is the only one conducted in the country and has implications beyond its borders. The third point highlights the significant disparities between quartiles on the basis of the relative effect of age (RAE), as well as the significant differences between sex and age. One notable advantage is that only a few publications have examined the relative age effect (RAE) with norm values in research conducted across different age groups, genders, and birth months. The findings of this study will be valuable for identifying children on the basis of the relative age effect (RAE) via the collected data, as well as incorporating other significant indicators to include talented youngsters in sports. Furthermore, it is crucial to incorporate the indications of the child’s growth, using the relative impact of age (RAE) as a normative reference; for example, we suggest it could be beneficial to have a TD system including percentile values as a good method for the evaluation of children with sports talent. In future research, it would be beneficial to incorporate additional protocols to examine the relative age effect (RAE), normative values, sex, age, and other general and specialized indicators. This would enhance the ability to cater to the needs of physical educators and sports professionals. Furthermore, it would be useful to encourage other studies that include more biomedical and human cross-sectional and multidisciplinary field samples of children, talent, athletes, and other actors in sports, on the basis of the relative age effect (RAE) with norm values, to present good practices and useful models for a more capable program for the identification and development of children with sports talent, including many other components such as priorities in the environment of children with sports talent as well as factors and influences on cultural values, to identify the social relationship between peers and those with potential for sports according to their talent [79,87]. It is appropriate to monitor additional physical performance indicators and examine their correlations with the relative age effect (RAE) in relation to normative values for chronological and biological age, gender, and level of competition among both sports participants and nonselected children. The application of new strategies in teaching can help children with sports talent through additional activities and extracurricular programs with the aim of further improving the potential of those children with sports talent, as well as those with the potential to follow a program to develop talent in sports. Including a transdisciplinary approach when working with children with sports talent during their identification and development, in which the areas of development could include the epigenetic, social, and psychological factors of the field of sports medicine, may help disentangle environmental influences on gene expression while following a program with gifted children [88,89,90].

## 5. Conclusions

This study examines the relative age effect (RAE) on anthropometric parameters and physical fitness tests in children aged 9–11 years, using normative values. The study also investigated any significant differences between the sexes. This study revealed that the impact of age on anthropometric parameters and physical fitness in 9-year-old children is greater for males born in the third quarter (Q3), whereas females born in the first quarter (Q1) are significantly different from males and those in the other quartiles (Q2, Q3, and Q4). Among 10-year-old children, males born in the second quartile (Q2) have an advantage over females born in the first quartile (Q1). There are significant variations between Q1, Q2, Q3, and Q4, which are more pronounced than the differences between males and females. At the age of 11, children born in the fourth quarter, regardless of gender, exhibit benefits compared with children born in other quarters. These advantages are shown both between sexes and among different quartiles. As shown graphically using the JAMOVI program, there are significant differences in anthropometric parameters between age (9, 10, 11) and gender, while relative age effect (RAE) and gender have not shown significant differences. In addition, there were no significant differences in physical fitness between age (9, 10, 11) and gender, while there was a significant difference between the relative age effect (RAE) and the static strength indicator. These advantages are shown both between sexes and among different quartiles. Furthermore, conducting long-term studies that encompass both sample size and length would be beneficial in confirming the impact of a program. Additionally, multidimensional studies that track anthropometric characteristics and physical fitness would provide valuable insights.

## Figures and Tables

**Table 1:** Basic anthropometric statistical characteristics and physical fitness tests at the ages of 9–11 years: male (626) and female (559).

Age	9		10		11	
Gender	Female	Male	Female	Male	Female	Male
Number	184	213	183	195	192	218
Mean ± Std.Dev	Mean ± Std.Dev	Mean ± Std.Dev	Mean ± Std.Dev	Mean ± Std.Dev	Mean ± Std.Dev	Mean ± Std.Dev
Height (cm)	135.8 ± 7.0	136.9 ± 6.6	141.4 ± 7.4	141.4 ± 6.9	148.0 ± 6.7	146.7 ± 7.1
Body mass (kg)	32.5 ± 34.4	34.4 ± 20.7	36.1 ± 9.1	37.7 ± 10.3	42.3 ± 11.2	41.0 ± 9.8
Body mass index-BMI	17.4 ± 3.5	17.5 ± 3.4	18.2 ± 3.7	18.5 ± 3.7	19.2 ± 4.2	18.9 ± 3.5
Flamingo balance test (cm)	16.8 ± 21.0	20.0 ± 25.1	20.5 ± 23.5	21.0 ± 25.3	21.3 ± 0.5	4.4 ± 2.8
Sit and reach (cm)	2.6 ± 0.3	0.9 ± 5.6	1.5 ± 5.6	0.7 ± 5.4	1.0 ± 10.5	−1.1 ± 5.4
Standing long jump (cm)	103.6 ± 18.2	117.1 ± 22.8	109.4 ± 22.1	124.9 ± 25.4	114.6 ± 24.1	130.3 ± 24.4
Countermovement jump (cm)	19.2 ± 3.7	21.2 ± 4.3	19.7 ± 6.3	20.6 ± 6.7	20.9 ± 4.7	22.7 ± 4.8
Handgrip strength (kg)	5.1 ± 4.9	5.9 ± 5.4	6.4 ± 6.2	7.9 ± 7.2	10.9 ± 8.2	10.8 ± 8.6
Sit ups (s)	11.9 ± 5.2	24.1 ± 4.8	12.8 ± 4.6	15.1 ± 5.4	13.5 ± 5.1	16.5 ± 5.5
20 m sprint (s)	4.7 ± 0.5	4.4 ± 0.4	4.6 ± 0.5	4.3 ± 0.5	4.5 ± 0.5	4.4 ± 2.6
Bent arm hang test (s)	4.55 ± 4.8	8.0 ± 8.2	5.8 ± 6.2	8.2 ± 9.3	5.1 ± 6.5	7.2 ± 8.3
10 × 5 m shuttle run (s)	26.0 ± 24.1	24.1 ± 3.0	24.7 ± 3.8	23.6 ± 3.1	25.0 ± 3.4	23.4 ± 3.1

**Table 2:** Relative age effect (RAE) and gender differences in percentages of females and males aged 9 years.

Gender			Females				Males				Q1, 2, 3, 4	M/F
Quartile			Q1	Q2	Q3	Q4	Q1	Q2	Q3	Q4	*p* Value	
Height (cm)	Very poor	5	125.0	126.0	126.0	120.0	124.7	127.9	127.8	123.5	*p* < 0.008 *	*p* < 0.819
		10	130.0	128.8	127.2	125.1	132.0	129.9	130.0	125.7		
	Poor	25	131.0	130.0	131.0	132.0	134.0	135.5	134.3	133.2		
	Average	50	138.0	140.0	135.0	136.0	137.5	141.5	137.7	137.5		
	Good	75	142.5	143.0	141.7	140.5	142.2	144.0	142.7	142.7		
	Very good	90	150.0	148.8	146.0	146.8	148.0	148.1	146.6	147.4		
	Excellent	95	156.5			149.4	151.0	151.2	153.3			
Body mass (kg)	Very poor	5	23.0	21.0	22.7	22.4	22.7	23.7	25.7	23.0	*p* < 0.170	*p* < 0.103
		10	24.0	22.4	24.1	23.3	24.7	25.9	26.5	23.5		
	Poor	25	27.8	26.2	26.9	26.5	27.5	30.0	28.0	26.3		
	Average	50	31.7	31.9	31.0	29.2	32.0	36.1	34.2	32.9		
	Good	75	39.0	38.3	40.3	32.6	38.0	42.3	42.5	41.5		
	Very good	90	49.0	54.4	51.5	53.3	46.6	52.6	49.0	44.6		
	Excellent	95	61.1		55.0		51.4	68.3				
Body mass index (BMI)	Very poor	5	13.6	12.4	13.5	14.1	13.4	13.8	14.3	13.2	*p* < 0.530	*p* < 0.253
		10	14.0	13.2	14.2	14.1	14.0	14.6	14.8	13.6		
	Poor	25	15.3	15.0	15.3	14.5	14.9	15.9	15.4	15.0		
	Average	50	16.8	17.3	17.0	16.4	16.7	17.7	17.3	17.4		
	Good	75	19.0	18.8	21.7	18.7	19.6	21.8	20.8	20.1		
	Very good	90	23.9	25.2	22.9	24.6	24.3	25.3	23.09	21.3		
	Excellent	95	26.6		27.3		25.0	30.9	25.4			
Flamingo Balance test (s)	Very poor	5	1	1	1	1	1	1	1	0.8	*p* < 0.105	*p* < 0.090
		10	1	1	2	1	1	1.3	2	0.9		
	Poore	25	2	2	2	6	3	2	5	4		
	Averge	50	6	3	11	19	10	6	12	17		
	Good	75	23	16	28	51.5	25	28	21	27		
	Very Good	90	45	67	51	74	41	48	64	45		
	Excellent	95	56		74		96	60	65			
Sit and reach (cm)	Very poor	5	−9.0	−9.0	−6.30	−8.0	−9.5	−7.6	−9.0	−11.0	*p* < 0.015 *	*p* < 0.780
		10	−9.0	−8.3	−5.60	−7.4	−6.0	−5.1	−9.0	−10.4		
	Poor	25	−2.5	−6.5	−3.00	−6.0	−3.0	−2.0	−3.7	−6.7		
	Average	50	5.0	0.5	4.00	−3.0	1.0	−1.0	1.5	5.5		
	Good	75	9.5	6.5	6.00	6.0	5.0	3.0	4.0	10.7		
	Very good	90	11.0	11.6	7.00	8.2	8.0	5.1	8.2	14.4		
	Excellent	95	15.0		8.50		10.0	8.2	13.8			
Standing long jump (cm)	Very poor	5	74.0	66.0	70.7	69.4	77.5	70.1	79.2	87.0	*p* < 0.000 **	*p* < 0.000 **
		10	82.1	66.9	79.9	70.5	89.0	77.3	86.6	87.9		
	Poor	25	95.2	81.5	96.3	83.9	103.2	90.0	102.7	95.1		
	Average	50	102.0	100.4	102.8	105.5	120.0	113.5	118.5	101.5		
	Good	75	116.8	119.2	106.4	126.0	133.0	133.2	140.0	112.0		
	Very good	90	129.0	123.6	122.8	146.3	140.5	145.6	152.8	140.3		
	Excellent	95	137.6		132.2		152.1	150.5	163.9			
Countermovement jump (CMJ)	Very poor	5	14.25	16.9	12.	14.2	12.20	13.4	15.0	14.9	*p* < 0.001 *	*p* < 0.001 *
		10	15.4	17.5	14.5	16.1	14.2	17.4	15.6	15.2		
	Poor	25	17.2	18.4	15.7	19.6	17.6	18.6	18.6	18.0		
	Average	50	19.0	20.0	19.7	21.0	21.2	20.5	21.5	19.8		
	Good	75	21.5	22.1	22.0	21.9	25.2	24.5	24.6	21.7		
	Very good	90	24.7	25.5	25.0	27.1	27.7	27.3	26.2	24.9		
	Excellent	95	28.8		26.6		30.4	27.8	28.0			
Handgrip strength (kg)	Very poor	5	0.50	1.00	0.0	1.0	0.50	1.0	0.0	1.0	*p* < 0.157	*p* < 0.001 *
		10	1.00	1.70	1.0	1.3	1.0	1.9	0.9	1.3		
	Poor	25	2.00	2.00	2.0	2.00	2.0	3.0	2.0	2.2		
	Average	50	4.00	3.00	4.0	4.0	4.0	4.5	5.0	6.5		
	Good	75	7.00	6.00	7.0	60.0	7.0	6.0	11.0	8.0		
	Very good	90	15.00	16.20	16.4	9.4	14.0	12.1	15.2	13.4		
	Excellent	95	15.50		18.0		19.0	14.1	17.0			
20 m sprint (s)	Very poor	5	5.69	5.27	5.5	5.2	5.6	5.4	5.2	5.2	*p* < 0.001 *	*p* < 0.164
		10	5.30	4.91	5.3	4.7	5.0	5.1	5.0	4.8		
	Poor	25	4.80	4.61	4.9	4.5	4.5	4.7	4.5	4.5		
	Average	50	4.47	4.29	4.5	4.2	4.2	4.3	4.3	4.2		
	Good	75	4.26	4.05	4.2	4.0	4.0	4.2	4.0	4.0		
	Very good	90	4.10	4.03	4.0	4.0	3.8	4.0	3.7	3.9		
	Excellent	95	3.97		3.7		3.6	3.9	3.7			
Sit ups (s)	Very poor	5	2.00	5.00	3.7	2.0	7.0	2.0	7.0	5.0	*p* < 0.000 **	*p* < 0.000 **
		10	5.00	5.70	4.4	2.6	8.0	4.7	7.0	6.5		
	Poor	25	10.50	9.00	11.0	7.0	12.0	12.0	11.2	14.2		
	Average	50	14.00	10.50	15.0	11.0	15.0	15.0	15.0	16.5		
	Good	75	16.00	13.00	19.0	15.0	17.0	20.0	18.0	21.50		
	Very good	90	20.00	14.30	21.2	22.4	19.0	21.1	22.4	24.4		
	Excellent	95	21.50		23.0		20.0	22.5	28.2			
Bent arm hang (s)	Very poor	5	0.00	0.00	0.0	0.0	0.00	0.0	0.0	0.8	*p* < 0.000 **	*p* < 0.004 *
		10	0.00	0.00	0.7	0.0	0.0	0.0	0.0	0.8		
	Poor	25	0.00	0.07	1.3	2.8	1.7	1.0	1.3	1.3		
	Average	50	3.21	2.88	2.3	3.9	6.0	2.8	5.4	3.2		
	Good	75	8.11	7.94	5.3	4.9	10.5	8.0	9.9	6.0		
	Very good	90	10.13	11.41	9.0	17.0	19.4	13.0	21.6	8.2		
	Excellent	95	12.54		10.3		25.2	19.0	28.4			
10 × 5 m meter shuttle run (s)	Very poor	5	32.98	29.16	34.6	30.4	28.5	30.2	32.2	29.9	*p* < 0.000 **	*p* < 0.000 **
		10	27.41	28.37	29.3	26.3	25.8	29.1	26.9	25.4		
	Poor	25	25.85	25.14	27.7	24.9	25.0	26.3	25.9	24.5		
	Average	50	24.39	22.58	25.5	22.6	22.8	24.3	23.3	26.3		
	Good	75	22.24	21.19	24.2	21.3	21.1	22.8	20.9	19.0		
	Very good	90	21.27	21.08	22.1	21.1	19.5	20.1	19.9	18.4		
	Excellent	95	20.59		20.4		18.9	18.9	18.2			

Legend: Percentages include very poor (5%, 10%), poor (25%), average (50%), good (75%), very good (80%), and excellent (90%). The first quartile (Q1) encompasses the months of January, February, and March. The second quartile (Q2) includes the months of April, May, and June. The third quartile (Q3) comprises the months of July, August, and September. Finally, the fourth quartile (Q4) encompasses the months of October, November, and December. Probability level: <0.001, **; <0.005, *.

**Table 3:** Relative age effect (RAE) and gender differences in percentages of females and males aged 10 years.

Gender			Females				Males				Q1, 2, 3, 4	M/F
Quartile			Q1	Q2	Q3	Q4	Q1	Q2	Q3	Q4	Sig.	
Height (cm)	Very poor	5	127.46	123.50	131.74	130.85	130.20	125.72	131.00	129.92	*p* < 0.979	*p* < 0.969
		10	131.64	131.60	132.54	132.80	132.68	135.00	132.55	131.72		
	Poor	25	137.00	135.00	136.40	137.50	135.40	137.00	136.00	138.00		
	Average	50	142.00	141.90	138.80	142.10	140.50	142.00	140.00	141.500		
	Good	75	147.75	146.50	144.40	146.95	144.55	146.00	146.80	145.00		
	Very good	90	151.90	153.60	150.00	149.56	149.92	151.00	151.08	150.20		
	Excellent	95	154.00	154.00	154.30	153.40	150.00	153.90	153.39	153.58		
Body mass (kg)	Very poor	5	23.11	25.00	26.07	24.96	27.00	25.20	25.66	25.28	*p* < 0.118	*p* < 0.294
		10	24.08	25.70	27.00	28.40	27.08	28.02	27.05	27.020		
	Poor	25	30.00	29.0	28.00	30.50	29.50	30.50	29.00	31.00		
	Average	50	34.45	32.00	34.05	36.00	35.90	34.60	34.00	36.00		
	Good	75	40.80	40.70	42.12	39.85	40.35	48.10	41.80	43.90		
	Very good	90	48.90	53.000	54.30	48.40	45.46	57.06	48.35	52.68		
	Excellent	95	56.00	61.00	55.32	55.80	59.00	65.90	56.04	54.84		
Body mass index (BMI)	Very poor	5	13.50	13.70	14.00	14.41	14.50	14.52	14.02	13.97	*p* < 0.431	*p* < 0.724
		10	14.29	14.42	14.36	14.64	14.84	14.74	14.53	14.88		
	Poor	25	15.54	15.26	15.16	15.75	15.21	15.36	15.49	16.15		
	Average	50	17.02	16.55	17.64	17.60	18.30	17.25	16.94	17.72		
	Good	75	20.35	19.50	21.39	19.85	20.39	23.46	21.15	20.78		
	Very good	90	22.75	26.27	24.46	23.56	22.95	25.50	22.35	23.37		
	Excellent	95	24.83	27.05	27.63	24.87	26.41	28.97	23.61	25.20		
Flamingo balance test (s)	Very poor	5	1	1	1	1	1	1	0.7	1	*p* < 0.818	*p* < 0.743
		10	1	2	1	1	1	1	1	2		
	Poor	25	3	3	3	4	4	3	2	4		
	Average	50	10	9	12	11	9	9	10	10		
	Good	75	26	25	32	23	26	39	30	34		
	Very good	90	61	46	62	57	50	63	64	62		
	Excellent	95	98	52	70	69	97	94	72	69		
Sit and reach (cm)	Very poor	5	−8.95	−6.00	−7.68	−6.30	−9.00	−7.40	−9.00	−8.00	*p* < 0.153	*p* < 0.308
		10	−7.80	−5.00	−6.65	−6.00	−6.00	−7.00	−7.09	−7.00		
	Poor	25	−2.00	−1.00	−4.00	−2.50	−3.55	−3.00	−3.00	−3.00		
	Average	50	2.00	1.00	2.00	1.00	1.20	0.00	1.00	2.00		
	Good	75	6.00	4.00	5.2	5.50	4.25	5.00	3.25	4.00		
	Very good	90	8.90	9.00	9.65	10.00	9.40	8.60	10.85	7.00		
	Excellent	95	12.00	12.00	12.65	11.20	10.18	10.40	11.45	10.00		
Standing long jump (cm)	Very poor	5	73.25	79.00	70.15	72.90	78.70	86.20	82.70	88.20	*p* < 0.000 **	*p* < 0.000 **
		10	87.00	90.00	78.80	79.44	86.28	90.52	89.88	94.60		
	Poor	25	94.92	105.00	90.75	90.50	111.40	112.0	104.82	105.00		
	Average	50	111.50	114.00	102.00	104.00	129.00	128.00	122.50	123.00		
	Good	75	129.62	127.00	123.75	120.00	149.50	146.70	136.42	142.00		
	Very good	90	140.16	132.00	138.72	127.88	158.58	160.00	153.91	156.88		
	Excellent	95	149.80	137.50	149.74	136.80	164.70	171.20	172.45	169.60		
Countermovement jump (CMJ)	Very poor	5	7.71	13.00	5.70	10.41	6.10	11.28	9.87	4.80	*p* < 0.054	*p* < 0.300
		10	9.08	13.30	7.00	13.84	7.78	13.58	12.11	10.00		
	Poor	25	16.42	15.00	15.17	17.75	16.00	18.50	17.05	16.90		
	Average	50	20.05	20.00	19.95	20.90	19.40	22.10	20.80	22.20		
	Good	75	24.05	22.20	22.62	23.40	26.95	24.40	24.17	25.21		
	Very good	90	28.70	24.50	26.40	29.30	30.90	28.80	27.98	28.48		
	Excellent	95	28.90	27.00	30.62	32.70	31.90	31.4	28.79	33.92		
Handgrip strength (kg)	Very poor	5	1.00	0.00	0.00	0.00	0.10	1.00	0.00	1.00	*p* < 0.032	*p* < 0.064
		10	1.00	0.00	1.00	1.00	1.20	1.00	1.00	2.00		
	Poor	25	2.00	2.00	2.00	1.50	3.00	3.00	2.75	3.00		
	Average	50	4.00	6.00	5.00	3.00	6.00	5.00	5.00	6.00		
	Good	75	7.00	12.00	11.50	6.00	7.50	14.00	12.50	12.00		
	Very good	90	15.80	15.00	15.50	11.40	14.00	22.60	18.00	19.20		
	Excellent	95	26.85	20.00	24.25	17.10	17.00	26.80	23.60	28.00		
20 m sprint (s)	Very poor	5	5.49	5.19	5.74	5.47	5.92	5.40	5.22	5.37	*p* < 0.000 **	*p* < 0.000 **
		10	5.21	5.12	5.4	5.1	5.13	5.06	4.90	5.04		
	Poor	25	4.96	4.75	5.07	4.84	4.76	4.58	4.64	4.60		
	Average	50	4.60	4.51	4.69	4.70	4.25	4.37	4.27	4.3		
	Good	75	4.36	4.28	4.31	4.40	3.96	4.0	4.05	3.97		
	Very good	90	4.01	4.16	3.94	4.17	3.79	3.78	3.98	3.80		
	Excellent	95	3.92	4.03	3.83	4.08	3.60	3.68	3.85	3.63		
Sit ups (s)	Very poor	5	5.00	6.00	3.35	1.8	4.30	3.80	3.55	4.00	*p* < 0.000 **	*p* < 0.000 **
		10	5.00	9.00	7.70	5.40	8.00	8.00	6.20	7.40		
	Poor	25	10.00	10.00	10.75	10.50	10.00	12.00	12.00	11.00		
	Average	50	12.00	13.00	13.00	15.00	15.00	17.00	16.00	15.00		
	Good	75	16.00	15.00	16.00	16.00	19.50	20.00	20.00	19.00		
	Very good	90	18.90	19.00	20.00	19.00	21.80	22.80	21.00	21.00		
	Excellent	95	20.95	21.00	21.65	20.00	22.90	24.40	23.45	23.00		
Bent arm hang (s)	Very poor	5	0.33	0.00	0.000	0.00	0.00	0.00	0.18	0.00	*p* < 0.003 *	*p* < 0.012 *
		10	1.09	0.50	0.00	0.1920	0.18	0.00	1.10	0.00		
	Poor	25	2.030	2.28	2.19	1.43	2.40	2.01	2.79	3.52		
	Average	50	4.39	5.75	4.38	4.08	5.23	4.33	4.46	5.23		
	Good	75	7.15	8.04	7.74	6.45	13.16	9.40	8.42	11.60		
	Very good	90	14.43	13.08	13.90	12.49	21.80	22.40	14.10	24.23		
	Excellent	95	21.85	15.55	17.59	13.99	24.10	33.20	29.34	28.10		
10 × 5 m shuttle run (s)	Very poor	5	29.82	28.91	32.70	31.80	30.16	29.27	29.12	29.00	*p* < 0.003 *	*p* < 0.072
		10	28.50	28.82	30.71	29.96	28.70	26.77	27.47	27.34		
	Poor	25	26.29	26.63	28.67	28.33	26.41	24.42	26.73	25.65		
	Average	50	25.16	24.34	26.49	25.50	23.43	22.90	24.12	23.41		
	Good	75	21.82	21.80	21.81	21.94	20.66	21.58	21.80	21.79		
	Very good	90	19.38	20.56	20.20	20.66	18.89	20.40	20.06	19.67		
	Excellent	95	16.80	17.61	18.77	19.06	18.62	20.01	19.36	18.84		

Probability level: <0.001, **; <0.005, *.

**Table 4:** Relative age effect (RAE) and gender differences in percentages of females and males aged 11 years.

Gender			Females				Males				Q1, 2, 3, 4	M/F
Quartile			Q1	Q2	Q3	Q4	Q1	Q2	Q3	Q4	Sig.	
Height (cm)	Very poor	5	137.0	137.6	135.2	134.9	134.3	134.6	133.9	136.0	*p* < 0.059	*p* < 0.009 *
		10	140.2	142.0	136.0	142.8	139.0	136.4	136.8	137.6		
	Poor	25	144.2	143.4	141.0	145.0	142.6	142.0	140.3	141.1		
	Average	50	149.0	148.8	145.7	149.0	148.0	147.5	145.2	145.2		
	Good	75	154.1	152.6	149.0	153.6	152.6	153.0	150.6	149.5		
	Very good	90	158.3	155.8	155.0	158.6	157.0	156.0	155.5	154.8		
	Excellent	95	159.1	165.0	156.1	161.5	159.6	158.0	160.1	158.8		
Body mass (kg)	Very poor	5	28.0	29.5	24.9	28.7	30.0	28.3	27.4	28.5	*p* < 0.212	*p* < 0.112
		10	32.5	29.8	26.0	31.7	31.0	29.1	29.9	30.0		
	Poor	25	36.2	34.7	31.2	36.1	34.1	32.7	33.3	32.9		
	Average	50	41.4	39.85	36.2	41.0	40.0	38.2	38.9	38.2		
	Good	75	49.2	47.7	42.9	55.8	46.7	49.5	48.4	44.6		
	Very good	90	62.3	54.9	57.3	62.8	52.6	58.1	56.9	50.8		
	Excellent	95	64.1	56.4	70.0	68.0	54.6	60.2	61.2	61.5		
Body mass index (BMI)	Very poor	5	14.6	13.95	13.4	14.4	14.5	14.4	14.5	14.9	*p* < 0.471	*p* < 0.771
		10	15.1	14.4	14.0	15.3	14.9	14.9	14.6	15.2		
	Poor	25	16.7	16.8	14.8	16.5	16.1	15.9	16.5	16.2		
	Average	50	18.6	17.93	17.3	19.2	18.5	17.7	18.3	17.2		
	Good	75	21.5	21.1	19.7	23.1	21.1	21.4	21.5	20.1		
	Very good	90	24.9	23.8	25.1	27.9	22.7	25.2	24.6	23.1		
	Excellent	95	28.9	26.5	30.4	28.9	24.2	27.0	25.8	26.0		
Flamingo balance test (s)	Very poor	5	1	1	1	2	1	1	1	1	*p* < 0.134	*p* < 0.133
		10	1	1	2	2	2	2	2	2		
	Poor	25	3	3	3	4	7	4	3	3		
	Average	50	8	6	7	15	20	12	12	14		
	Good	75	28	17	22	39	45	32	30	27		
	Very good	90	66	42	51	59	87	92	58	44		
	Excellent	95	92	72	52.8	83.2	88	98	86	59		
Sit and reach (cm)	Very poor	5	−10.0	−9.0	−7.3	−10.0	−10.0	−12.4	−9.0	−9.0	*p* < 0.008 *	*p* < 0.008 *
		10	−9.0	−9.0	−7.0	−10.0	−9.0	−9.0	−8.0	−8.0		
	Poor	25	−2.7	−6.2	−3.0	−4.5	−6.0	−4.0	−5.2	−5.0		
	Average	50	2.0	1.8	1.6	5.0	−2.5	1.0	0.0	−2.0		
	Good	75	5.7	5.5	5.0	8.5	2.2	4.5	3.0	2.0		
	Very good	90	9.3	12.4	8.0	13.0	6.0	7.0	4.0	6.0		
	Excellent	95	14.1	13.8	12.0	15.8	8.1	9.4	6.2	7.0		
Standing long jump (cm)	Very poor	5	59.3	77.3	75.2	93.8	88.0	92.9	100.5	77.9	*p* < 0.000 **	*p* < 0.000 **
		10	78.5	81.9	83.7	96.0	100.0	99.4	106.9	89.3		
	Poor	25	98.0	96.0	97.0	100.0	117.6	118.0	116.0	107.2		
	Average	50	119.1	106.7	115.0	121.0	131.0	130.0	129.5	124.5		
	Good	75	133.0	119.3	125.3	145.5	152.8	146.0	145.3	144.8		
	Very good	90	147.9	141.2	135.7	161.4	163.1	168.0	161.1	165.1		
	Excellent	95	153.0	158.2	147.0	165.3	168.9	174.8	166.1	175.8		
Countermovement jump (CMJ)	Very poor	5	12.0	14.1	12.0	14.3	15.8	15.7	13.1	14.8	*p* < 0.000 **	*p* < 0.000 **
		10	13.5	16.2	15.0	15.5	17.0	16.9	15.6	15.5		
	Poor	25	17.0	17.9	17.9	18.4	20.3	19.0	18.4	18.9		
	Average	50	21.2	19.1	21.3	21.1	21.9	21.5	22.5	23.0		
	Good	75	24.3	23.9	22.6	26.1	25.1	26.5	26.4	26.8		
	Very good	90	26.9	28.3	25.4	29.2	28.2	30.4	29.7	29.6		
	Excellent	95	28.3	30.8	27.2	30.4	31.1	33.7	32.6	31.0		
Handgrip strength (kg)	Very poor	5	1.0	2.1	1.0	1.4	2.0	1.0	1.0	1.1	*p* < 0.855	*p* < 0.855
		10	1.0	3.3	2.0	2.8	2.7	2.0	2.9	2.0		
	Poor	25	3.0	5.7	5.0	4.5	5.0	4.0	5.7	5.0		
	Average	50	7.0	9.0	7.0	13.	7.0	6.0	10.0	6.0		
	Good	75	14.7	14.2	13.0	21.0	15.0	13.0	19.2	15.2		
	Very good	90	20.0	21.0	20.7	30.2	25.3	21.8	28.0	21.7		
	Excellent	95	24.9	22.8	24.1	34.8	28.0	30.4	32.1	25.0		
20 m meter sprint	Very poor	5	6.5	5.5	5.4	5.2	4.8	5.2	5.3	5.6	*p* < 0.454	*p* < 0.455
		10	5.3	5.3	5.3	4.9	4.7	4.9	5.0	4.8		
	Poor	25	4.9	5.0	4.9	4.6	4.5	4.6	4.4	4.4		
	Average	50	4.5	4.5	4.5	4.4	4.1	4.3	4.2	4.2		
	Good	75	4.1	4.2	4.2	4.0	4.0	3.9	3.9	4.0		
	Very good	90	3.8	4.0	4.0	3.8	3.7	3.6	3.7	3.7		
	Excellent	95	3.7	3.8	3.9	3.7	3.5	3.5	3.5	3.6		
Sit ups (s)	Very poor	5	3.8	6.0	3.6	3.9	7.3	3.8	6.9	4.1	*p* < 0.000 **	*p* < 0.000 **
		10	7.0	6.0	5.0	7.8	10.0	7.0	10.0	6.6		
	Poor	25	11.2	9.7	9.0	10.5	13.0	14.0	15.0	11.5		
	Average	50	14.0	12.0	14.0	14.0	16.0	17.0	17.0	16.0		
	Good	75	18.0	17.0	17.0	17.5	19.0	21.0	20.0	20.2		
	Very good	90	20.0	18.7	20.0	20.2	22.0	24.2	23.2	27.0		
	Excellent	95	20.1	19.8	23.7	22.1	26.6	25.6	27.0	28.0		
Bent arm hang (s)	Very poor	5	0.0	0.0	0.0	0.0	0.0	0.0	0.0	0.0	*p* < 0.986	*p* < 0.987
		10	0.0	0.3	0.0	0.0	0.4	0.0	0.4	0.0		
	Poor	25	1.3	1.3	1.2	1.5	1.7	2.9	1.9	1.2		
	Average	50	2.2	3.2	2.6	3.4	5.1	5.3	3.2	4.1		
	Good	75	5.2	7.4	5.4	9.0	11.5	11.4	8.5	8.3		
	Very good	90	12.9	13.4	14.1	12.9	18.7	20.1	19.3	13.0		
	Excellent	95	17.1	34.6	20.4	18.1	37.6	26.9	23.2	16.5		
10 × 5 m shuttle run (s)	Very poor	5	30.8	32.2	31.3	30.9	27.3	29.4	29.1	30.4	*p* < 0.000 **	*p* < 0.000 **
		10	30.0	30.0	29.4	29.7	25.7	28.4	28.0	27.8		
	Poor	25	28.0	27.0	27.2	26.1	24.2	25.9	26.6	25.5		
	Average	50	24.8	24.9	25.3	23.9	22.4	22.2	24.6	23.8		
	Good	75	22.5	21.9	23.3	21.4	20.3	20.5	21.9	21.7		
	Very good	90	20.4	20.8	21.7	20.4	18.3	19.3	20.7	19.3		
	Excellent	95	19.6	20.4	20.4	19.4	17.1	18.7	20.4	18.6		

Probability level: <0.001, **; <0.005, *.

## Data Availability

The original contributions presented in this study are included in the article; further inquiries can be directed to the first and corresponding author.
